# Bahamas National Implementation Project: Proposal for Sustainability of an Evidence-based HIV Prevention Intervention in a School Setting

**DOI:** 10.2196/14816

**Published:** 2020-08-21

**Authors:** Bo Wang, Lynette Deveaux, Sonja Lunn, Veronica Dinaj-Koci, Samiran Ghosh, Xiaoming Li, Sharon Marshall, Glenda Rolle, Nikkiah Forbes, Bonita Stanton

**Affiliations:** 1 Department of Population and Quantitative Health Sciences University of Massachusetts Medical School Worcester, MA United States; 2 Office of HIV/AIDS Ministry of Health Nassau Bahamas; 3 Department of Family Medicine and Public Health Sciences Wayne State University School of Medicine Detroit, MI United States; 4 Department of Health Promotion, Education and Behavior Arnold School of Public Health University of South Carolina Columbia, SC United States; 5 Department of Pediatrics Division of Adolescent Medicine Children's Hospital of Michigan Detroit, MI United States; 6 Hackensack Meridian School of Medicine Seton Hall University Nutley, NJ United States

**Keywords:** adolescent, HIV prevention, implementation, sustainability, evidence-based intervention

## Abstract

**Background:**

Sustained implementation of school-based prevention programs is low. Effective strategies are needed to enhance both high-level implementation fidelity and sustainability of prevention programs.

**Objective:**

This proposed study aims to determine if the provision of either biweekly monitoring and feedback and site-based assistance and mentorship or both to at-risk and moderate-performing teachers with monitoring through an enhanced decision-making platform by the Ministry of Education (MOE) and Ministry of Health (MOH) based on the real-time implementation data will increase national implementation fidelity and result in sustained implementation over time.

**Methods:**

This study will target government schools including 200 grade 6 teachers in 80 primary schools and 100 junior/middle high school teachers (and their classes) on 12 Bahamian islands. Teacher and school coordinator training will be conducted by the MOE in year 1, followed by an optimization trial among teachers in the capital island. Informed by these results, an implementation intervention will be conducted to train using different levels of educational intensity all at-risk and moderate-performing teachers. Subsequently selected training and implementation strategies will be evaluated for the national implementation of Focus on Youth in the Caribbean and Caribbean Informed Parents and Children Together in years 2 to 5.

**Results:**

It is hypothesized that a more intensive training and supervision program for at-risk and moderate-performing teachers will enhance their implementation fidelity to the average level of the high-performing group (85%), an HIV prevention program delivered at the national level can be implemented with fidelity in grade 6 and sustained over time (monitored annually), and student outcomes will continue to be highly correlated with implementation fidelity and be sustained over time (assessed annually through grade 9). The proposed study is funded by the National Institute of Child Health and Human Development from August 1, 2018, through May 31, 2023.

**Conclusions:**

The study will explore several theory-driven implementation strategies to increase sustained teacher implementation fidelity and thereby increase the general public health impact of evidence-based interventions. The proposed project has potential to make significant contributions to advancing school-based HIV prevention research and implementation science and serve as a global model for the Fast Track strategy.

**International Registered Report Identifier (IRRID):**

PRR1-10.2196/14816

## Introduction

### Background

Despite great progress over the past three decades in the prevention and treatment of HIV/AIDS, the global HIV epidemic remains a major cause of morbidity and mortality. HIV has become a disease of the young, with 40% of new infections worldwide occurring among those aged younger than 25 years. AIDS-related deaths increased among adolescents by approximately 50% between 2005 and 2012. Children and adolescents are significantly less likely to receive treatment than adults (23% versus 38%) [1-3]. The United Nations (UN) General Assembly has identified the pivotal role of young adults in the epidemic and is calling for comprehensive, evidence-based prevention approaches [4].

There has been much progress in curtailing the global HIV epidemic, however. The UN’s Millennium Development Goals clearly articulate relevant target objectives with specific, trackable action plans and widely publicized annual progress reports [5]. The UN and nations across the world established Sustainable Development Goals including the ambitious but achievable goal of the Joint United Nations Programme on HIV/AIDS (UNAIDS) to end the AIDS epidemic as a global threat by the year 2030. This goal requires a 90% reduction of new HIV infections. Demographic groups that have not fully benefitted from the advances in controlling the epidemic will need to be reached. The blueprint for this goal is the UNAIDS Fast Track strategy which states that “[v]ery high levels of coverage for programmes that promote correct and consistent condom use will be needed in all types of epidemics [1].”

Critical to the success of Fast Track are identifying and addressing the issues involved in maintaining the effectiveness of evidence-based HIV prevention programs as they are transformed from research to practice [6-8]. Primary prevention refers to actions or information aimed at preventing the onset of illness before the disease process begins or improving health through changing the impact of social and economic determinants on health. Most HIV prevention programs are designed to promote abstinence and/or condom use to prevent disease transmission. For an intervention that has been demonstrated to be effective in a research setting to become an effective public health tool, it must be implemented and sustained and delivered in a fashion that is likely to reproduce its effectiveness (fidelity). These concepts have been the source of substantial scientific inquiry [9,10]. Research to date indicates that sustained implementation (delivery of some or all of the intervention over time) of evidence-based behavioral interventions is low [11]. Studies assessing fidelity of implementation of effective programs report considerable deviance from the original, evidence-based curricula [12]. However, while fidelity of implementation is critical to success [12-14], many studies have found that some adaptation is inevitable and may signal commitment to the new program [12,15,16] and/or strengthen the program’s effectiveness [17]. Substantial literature underscores the importance of contextual fit of the new intervention within the local culture [18] that may be achieved through adaptation. These findings signal the importance of local, culturally appropriate implementation efforts for the next stage of HIV research [18,19].

Over the past decade, multiple disciplines have embraced the importance of moving evidence-based interventions into community settings in order to benefit society [20]. Numerous implementation and dissemination models have been developed, including the Exploration, Preparation, Implementation, Sustainment model by Aarons et al [21]. This model offers several strengths (logical, evidence-based) and specifies variables that may play crucial roles at different phases in the implementation process, impacting the ultimate success of intervention delivery [21]. It appears to be well suited as the platform for the research questions to be addressed.

Although implementation of evidence-based interventions in the school setting remains low [20,22], efforts to do so are increasing. Five factors found to be important are community-level aspects including politics and funding, implementer (teacher) characteristics, characteristics of the intervention program itself including adaptability and compatibility with the local environment, organizational capacity (including support from the leadership), and training and ongoing support [18,23,24]. Activities and materials supporting these concepts (eg, curriculum manuals and videos) appear to enhance the success of implementation. The implementation approach Fidelity Through Informed Technical Assistance and Training [23], consistent with the Exploration, Preparation, Implementation, Sustainment model and our own work [25,26], addresses threats to implementation fidelity through monitoring of implementation data provided by teachers and observers. In one study, the use of the Fidelity Through Informed Technical Assistance and Training approach was associated with an overall 98% curricular adherence [23]. Factors inconsistent with these supporting elements (such as lack of school time, competing priorities) undermine implementation [27]. Growing literature supports the evidence base for the utility of a social support network of practitioners or teachers (communities of practice) [28], confronting similar implementation challenges in working together [29,30]. In this study, communities of practice refers to a group of teachers who have ongoing interaction around the implementation of an effective HIV intervention. This social support network provides an environment in which teachers can share their experiences and discuss their progress and challenges in implementing HIV interventions in schools. Despite such advances, it is still not known if the public health outcomes anticipated from broad implementation of evidence-based programs are occurring and/or are sustained over time [31].

In the 1990s, with an HIV seroprevalence of 4%, the Bahamas embarked on an interagency approach targeting Bahamian children and adolescents and involving the Bahamian Ministries of Health (MOH) and Education (MOE) to reverse the escalating rates of HIV [32]. Over the past two decades, Bahamas MOE and MOH and our research team have adapted a US Centers for Disease Control and Prevention “best evidence” HIV prevention program to produce the Focus on Youth in the Caribbean (FOYC) and Caribbean Informed Parents and Children Together (CImPACT) risk reduction programs to address the HIV epidemic in the Bahamas. Two randomized, controlled longitudinal trials of FOYC and FOYC+CImPACT found the programs to be effective in improving knowledge, condom-use skills, and/or self-reported risk behaviors. In 2010, the MOE included FOYC in the government grade 6 curriculum nationwide, with boosters in grades 7 and 8. The MOE now plans to expand the offering to the more effective but logistically more complex FOYC+CImPACT version.

The HIV prevalence rate in the Bahamas has been declining during the past 20 years. UNAIDS global AIDS monitoring found HIV prevalence among the general population to be 1.9% in 2017 (0.6% and 0.7% among young women and men aged 15 to 24 years, respectively) [33]. The Bahamas has been providing preexposure prophylaxis (PrEP) through the public health system since 2018 [33]. AIDS remains a leading cause of death among Bahamians aged 25 to 44 years [34].

In summary, data from the national implementation study conducted from 2011 to 2016 showed a strong, positive correlation between number of core activities delivered and positive student outcomes [14,25,26]. Nevertheless, only about 50% of core activities were delivered, consistent with the literature on implementation of school-based programs [12]. Our findings confirmed prior research indicating that sustained implementation is low and should be a research priority [35,36]. To address this challenge, research that explores several theory-driven implementation strategies (eg, innovative teacher training and support, implementation monitoring and feedback, role of a curriculum implementation committee) to increase sustained teacher implementation fidelity, and thereby increase the general public health impact of evidence-based interventions, is indicated.

### Study Aims

Reflecting this background, our ongoing research aims to determine if the provision of either biweekly monitoring and feedback (BMF) or site-based assistance and mentorship (SAM) or both through a community of practice to at-risk and moderate-performing teachers with monitoring through an enhanced decision-making platform by the MOE and MOH based on the real-time implementation data will increase national implementation fidelity and result in sustained implementation over time.

## Methods

### Study Overview

FOYC+CImPACT is being implemented in grade 6 by approximately 200 grade 6 teachers. Annual FOYC boosters for the students will be conducted by the junior high school Health and Family Life Education (HFLE) teachers (approximately 100). The research team includes the US researchers, the Bahamian research office, and the 45 school coordinators who will gather and transmit the data from the field to the research office. All decisions regarding implementation of FOYC+CImPACT are being made by the Bahamian MOE and MOH, but the researchers are available for consultation at any time and will be formally involved through the regularly scheduled Fast Track School-Term Implementation Committee (implementation committee) which has been designed specifically to integrate and coordinate the roles of data, operations, and decision making. The implementation committee will review the implementation data presented by the researchers and make decisions regarding the need for any changes in implementation of FOYC+CImPACT. The implementation committee will meet once per school term (3 times per year). Committee members will include representatives from the MOE, including those responsible for curriculum development for all subjects, and the MOH, including those responsible for the HIV prevention program. Inclusion of these high-level decision makers from the MOE and MOH in program rollout, monitoring, and decision making underscores the importance of the FOYC+CIMPACT training to the nation’s Fast Track agenda. Researchers will present a summary of data collected, implementation status including any modifications made, and analyses prior to each meeting. The committee will discuss progress, decide if any implementation strategies require change, and identify data and programmatic needs to maximize FOYC+CImPACT’s benefits to students and to the Fast Track initiative.

### Evidence-Based Approaches to Increase Teacher Implementation

The MOE will give all teachers a FOYC+CImPACT 24/7 flash drive for point-of-care guidance as they prepare the lessons [37]. The MOE will deploy its peer-mentoring program for FOYC+CImPACT. High-performing teachers will serve as team leaders and provide guidance and onsite assistance to low- and moderate-performing teachers to increase their skills and self-efficacy [22]. High-performing and at-risk teachers will be identified through real-time implementation monitoring [23] and a pretraining 7-question screening tool [38]. As the goal is to achieve and sustain at least 85% implementation compliance (the average performance of the high-performing teachers) [25,38], implementation will be monitored biweekly with feedback provided to the teachers as per MOE policy. Decisions regarding the need for change will be made by the implementation committee based on data.

### Measures

#### Measure Assessing Implementation and Student Outcome

We are using 9 measures and 1 student questionnaire (Health Risk and Protective Factors) that were developed and employed in our prior implementation study (Table 1), with some modifications (pilot-tested in the Bahamas) based on validated scales from school implementation studies [39-41]. The new scales include assessments of teacher autonomy (5 items), perceived principal supportiveness (4 items), teacher self-efficacy (3 items), teacher attitudes toward sex education in schools (8 items), and teacher confidence (5 items). These scales are included in teachers’ measures of impression before and after teaching and will be further tested in the proposed study. These measures will be administered to the teachers before and after implementing FOYC+CImPACT to assess factors influencing fidelity of intervention implementation.

**Table 1:** Flow of measures.

Measure	Description	Responsibility
Teacher checklist	The checklist includes the 30 activities contained in the FOYC^a^ curriculum. Teachers indicate which activities they have/have not taught in each session and record whether they have taught each activity as outlined in the manual or have modified it and their level of comfort in teaching each activity and student engagement	Teacher
Observation log	This measure mirrors the teacher checklist. Approximately 10% of FOYC and 20% of CImPACT^b^ classes for each teacher will be independently observed	School Coordinator
Workshop preevaluation	Assess whether all days of FOYC+CImPACT training were attended and perceptions before training and past experience about the curriculum	Teacher
Workshop postevaluation	Assess perceptions after training	Teacher
Impression before teaching	Assess factors influencing fidelity of intervention implementation including teacher perceptions of the importance of prevention programs, HIV prevention, and FOYC intervention; teacher confidence in teaching the FOYC intervention; teacher sense of ownership of the curriculum, and teacher education, years as a teacher, and training in interactive teaching	Teacher
Impression after teaching	Assess factors influencing fidelity of intervention implementation, teacher reasons for not being able to complete delivering the FOYC curriculum, and perceived student benefits from FOYC curriculum	Teacher
Workshop observer log	Assess training in the teacher training workshop given prior to teaching. These checklists assess whether each activity that should have been taught during the workshop was taught	Assistant project manager
School coordinator biweekly assessment	This measure is based on other real-time tracking measures assessing FOYC+CImPACT scheduling, teaching, and form-completion activities	School Coordinator
Programmatic assessment	This measure tracks program changes made by the senior education officers or the implementation committee	Project manager
HRPF^c^ final exam	Assessing student outcomes as a function of teacher implementation fidelity and sustainability thereof over time. The HRPF^c^ will be administered to the students prior to delivery of FOYC+CImPACT in grade 6 and at the end of the school year in grades 6, 7, and 8	Students

^a^FOYC: Focus on Youth in the Caribbean.

^b^CImPACT: Caribbean Informed Parents and Children Together.

^c^HRPF: Health Risk and Protective Factors.

#### Data Flow Management

Every teacher will be assigned to a school coordinator whose responsibility will be to collect the forms and return them biweekly by email, mail, in person, or carrier to the central data system at the research office. The school coordinator will also be responsible for completing the school coordinator biweekly assessment, which contains information about all scheduled activities and measures for each teacher assigned to her and their completion or otherwise. It is important to have this information independently observed and recorded so that the MOE and research team are able to determine the status of implementation at all times and the data forms are continually tracked. Project manager will review this data biweekly.

### Field Work: Program Implementation and Research

#### Training Workshop

In the first quarter of year 1, all members of the research team including the 45 school coordinators participated in a 1.5-day training workshop, beginning with an overview of the Fast Track initiative and the FOYC+CImPACT program, followed by roles, responsibilities, data flow, ethical issues, and practice with the forms. Procedure manuals were distributed. A 1-day refresher training will be conducted in subsequent years for new and existing personnel.

#### Group Mentoring

Based on the principles of communities of practice, the MOE created SAM, a 2-tiered group mentorship program, to deploy the strength of high-performing teachers to help teachers who are struggling.

- General guidance and biweekly meeting: one high-performing teacher per school will serve as team leader and provide guidance to low- and moderate-performing teachers with their preparation and planning of FOYC+CImPACT sessions. The team leader will meet all grade 6 teachers and HFLE teachers (the middle school teachers involved) weekly to discuss their progress, identify challenges teachers are experiencing, and provide tips and guidance during the meeting. The team leader will promote group activities and enhance interaction among teachers in these meetings.
- Onsite assistance and observation: at-risk or moderate-performing teachers will be invited to observe while a session is being taught by a high-performing teacher/team leader in the classroom.

An additional program, enhanced SAM, will be offered to teachers who still have difficulties in teaching sensitive topics. As part of enhanced SAM, these teachers will be observed in the classroom by the team leader who will provide onsite assistance. These strategies, consistent with the principles of communities of practice [29,30], have evolved as part of the Bahamian school system’s culture to support new/challenged teachers.

#### Multiphase Optimization Strategy Design-Based Trial

This trial examines the effect of each training/implementation component for at-risk and moderate-performing teachers and whether the presence or absence of a component has an impact on the performance of other components. An advantage of the multiphase optimization strategy (MOST) design is its ability to identify active components making significant contributions to the overall effect from those that are not doing so, hence not worth retaining. For our MOST trial, there are two intervention components, one with 2 levels (ie, BMF) and the other with 3 levels (ie, SAM), which corresponds to a full factorial with 6 experimental conditions (Table 2). We chose the teacher implementation checklist to identify high-performing teachers as a measure of success (optimization criteria). Using our 7-item preimplementation school screening tool [38], we identified teachers who are at risk for not implementing the FOYC intervention curriculum and moderate- and high-performing teachers. All at-risk teachers and moderate-performing teachers were invited to participate in a factorial experimentation, using a MOST trial design. Workshop/video was provided to all teachers and treated as a constant component in the experiment. At the beginning of the trial, at-risk and moderate-performing teachers were asked to attend a 2-day curriculum workshop and each received an educational video. Following the teacher training, teachers were randomly assigned to 1 of the 6 experimental conditions and asked to teach the intervention curriculum for 1 semester. The primary outcome is implementation fidelity as assessed through the teacher implementation checklist and observer form. This effectiveness information is used to decide which set of components to select for at-risk or moderate-performing teachers. By conducting the proposed optimization phase of the MOST trial in year 1, we are determining the role of BMF and SAM (including enhanced SAM) and/or their combinations with the training workshop in improving implementation fidelity. The optimized teacher training and implementation packages will be used to train all at-risk and moderate-performing teachers for the subsequent national implementation of FOYC+CImPACT (12 islands in years 2 to 5).

**Table 2:** Multiphase optimization strategy design-based trial: optimization of training and implementation strategies.

Experiment condition	Workshop/video	Biweekly monitoring and feedback	Site-based assistance and mentorship
1 (n=15)	Yes	Yes	No SAM^a^
2 (n=15)	Yes	Yes	SAM
3 (n=15)	Yes	Yes	Enhanced SAM
4 (n=15)	Yes	No	No SAM
5 (n=15)	Yes	No	SAM
6 (n=15)	Yes	No	Enhanced SAM

^a^SAM: site-based assistance and mentorship.

#### Programmatic Implementation Including Change

Each stage of implementation will be assessed and tracked via the 9 measures. National implementation by grade begins in year 2 with grade 6 (full program). This will be followed in subsequent years by the grade 7 (boosters) as well as continued grade 6 full program, etc, such that by year 5, all classes of grades 6 through 9 in the 115 government schools nationwide will be participating. National implementation will begin with the teacher workshops. After the workshops, teachers will begin the actual implementation in the classrooms, beginning with the FOYC sessions during regular HFLE class time and scheduling the CImPACT sessions at a time when parents can attend. Data regarding the teaching process will be measured, including teacher perceptions before and after teaching FOYC+CImPACT, teacher checklist of what was taught, and observation by the school coordinator. Teacher compliance and success in scheduling, conducting at least 85% of all activities, and completing all forms will be tracked in biweekly and programmic assessments by the school coordinator. The project manager will meet at least weekly with the national school coordinators to review and document on the programmatic assessments any changes made by MOE relevant to the implementation of FOYC+CImPACT. Three times per year, the implementation committee will review program progress. The research team will assemble, organize, and enter all of the data using the Autodata System’s Scannable Office.

### Statistical Analysis

#### Effectiveness of Training and Implementation Strategies

To test the main effects and interactions among intervention components, we will use a standard analysis of variance (ANOVA) using effect coding (for two level: –1 = no and +1 = yes; for three level: –1 = no SAM, 0 = SAM, and +1 = enhanced SAM) rather than dummy coding (0, 1 or 0, 1, 2, etc). Effect coding has several advantages over dummy coding. First, effect coding produces estimates of main effects and interactions that are consistent with the classic definitions of ANOVA effects [42]. Second, effect coding preserves power for interactions [43].

We will make a preliminary selection of components that have achieved main effects (exceeding statistical significance or demonstrating medium-to-large effect size). This preliminary selection will then be reevaluated in light of any substantial interaction effects that have been detected to gain an understanding of how the components work in combination. Depending on the optimization criteria identified (implementing at least 85% of core activities), this would then be combined with other information (eg, cost, feasibility, scalability) to make a final selection of components [44]. This information will guide assembly of an optimized implementation package that achieves target outcomes with the least resource consumption and participant burden.

#### Sustainability of Implementation

Percentages of core activities (30 core activities in FOYC and 5 core activities in CImPACT) completed by all grade 6 teachers will be computed to assess whether the teachers can implement the prevention program with fidelity (delivery of more than 85% of core activities). For grade 7 to 9 teachers, percentages of core activities in booster sessions completed with fidelity will be computed to assess implementation fidelity

To assess whether delivery is sustained over time, percentages of core activities completed with fidelity by grade 6 teachers among the next cohorts of grade 6 students (and among grades 7 to 9 teachers, percentage of core activities in the boosters among the next cohorts of students) will be computed and compared across the cohorts of student classes. Sustainability refers to teachers’ continued implementation of the intervention with fidelity (at least 50% of core activities) and adherence to program principles in 3 years (at 3 yearly follow-ups).

#### Student Outcomes

Bivariate analysis and mixed-effects modeling will be conducted to assess the relationship between implementation fidelity and student outcomes. In bivariate analysis, we will categorize the fidelity score into 3 levels: high fidelity (teachers complete more than 85% of core activities with fidelity), average fidelity (teachers complete 70% to 85% of core activities with fidelity), and low fidelity (teachers complete less than 70% of core activities with fidelity). The differences in grade 6 student outcomes at baseline and year-end follow-up across 3 levels of implementation fidelity and differences in the change scores between groups will be assessed using ANOVA (for HIV/AIDS knowledge, condom-use skills, perceptions) and Pearson chi-square tests (for self-reported behaviors). The test statistics (F score, chi-square) will be adjusted for the clustering effects of classroom and/or school using variance inflation factors.

The association of teacher implementation fidelity with student outcomes will be further examined using mixed-effects modeling (for knowledge, skills, and perceptions) and generalized linear mixed modeling (for self-reported behaviors), controlling for clustering effects of classroom/school, student age, sex, and baseline differences. Mixed-effects modeling analyses will be run using combined grade 6 teacher and grades 7 to 9 teacher fidelity score to assess overall effects of implementation fidelity of FOYC+CImPACT on student outcomes. Bivariate analysis and mixed-effects modeling will also be performed to assess the impact of different levels and types of curricular changes on student outcomes. To assess whether student improvements gained from grade 6 national implementation are sustained over time, a generalized estimating equation model will be used to examine the difference in student knowledge, skills, perceptions, and self-reported behaviors across the time points. Analyses will be performed using the SAS 9.4 (SAS Institute Inc) statistical software package.

**Figure 1:**
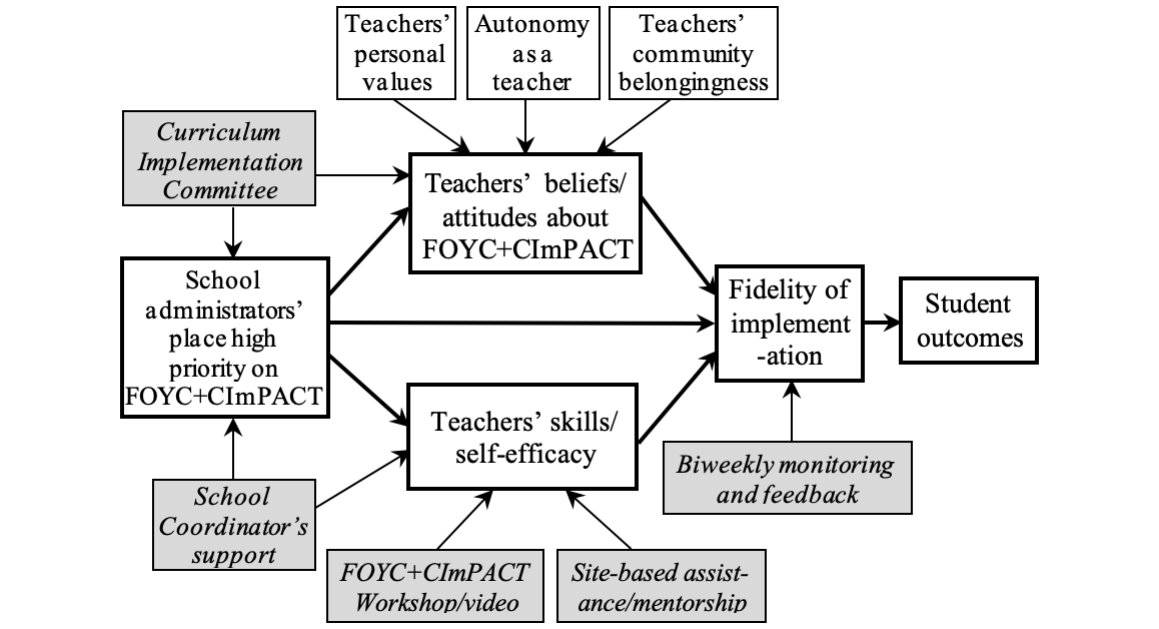
Hypothesized conceptual model showing relationships among factors that influence fidelity of implementation and student outcomes. The implementation strategies are indicated by shadowed/italicized constructs.

Structural equation modeling analysis will be conducted to examine the relationships among factors influencing teacher fidelity of implementation and student outcomes using the Mplus 8 (Muthen & Muthen). We developed a hypothetical conceptual model (Figure 1) based on a synthesis of the empirical literature and our implementation work [21,45,46]. The model posits that teacher attitudes toward the intervention and self-efficacy in teaching the curriculum have a direct effect on fidelity of implementation, which will impact student outcomes. Ongoing interest and support from the school administration will reinforce teacher perception that this program is a high priority for their school administrators and indeed the nation. The identification of high-performing teachers as mentors will empower these teachers by increasing their sense of autonomy and role in the community. Given the importance of self-efficacy in implementation fidelity, components have been added to the implementation effort to increase real and perceived self-efficacy (workshop/video and site-based assistance and mentorship) [22,45]. Biweekly monitoring and feedback are hypothesized to increase teacher fidelity of implementation. The curriculum implementation committee is expected to improve teacher and school administrator attitudes toward the intervention. A starting model will be estimated to investigate the interrelationships among factors influencing fidelity of implementation. A full model will be constructed by including student outcome latent variables in the revised fidelity model, using the cluster option in Mplus. Standardized regression coefficients for all paths will be estimated using robust maximum likelihood estimation. A good model fit is indicated when the standardized root mean square residual and root mean square error of approximation are less than 0.05 and comparative fit index is greater than 0.95 [47,48].

## Results

The research protocol R01 HD095765 (BW and BS) was funded by the Eunice Kennedy Shriver National Institute of Child Health and Human Development (NICHD) for the period August 1, 2018, through May 31, 2023. Notice of award was received on July 23, 2018. Enrollment and teacher training began in October 2018 and will continue through September 2021. As of April 2019, 98 grade 6 teachers from two islands who teach HFLE classes completed a 2-day teacher training workshop. Twenty-four school coordinators (including 2 national school coordinators) were identified and trained for the purpose of tracking teacher implementation and progress biweekly, collecting teacher measures, and identifying and reporting issues and problems to the research office, located in New Providence. Seventy-two at-risk and moderate-performing teachers in 24 schools in New Providence participated in the MOST design-based trial from February to April 2019.

Teachers teach HIV intervention as part of the HFLE curriculum. Teacher participation in the project is voluntary. Parents are advised that the course will be taught to the students and they can request that their children not participate in the teaching sessions. While rare, these requests are accommodated.

At the beginning of the project, a small number of parents expressed concerns about the FOYC curriculum. The project coordinator met with these parents and explained the benefits of the sexual risk reduction intervention among adolescents in a nation such as the Bahamas with a national HIV prevalence among adults of 1.9% in 2017. The team did advise the parents that they could request that their children not participate in the teaching sessions. However, after this discussion, the parents were no longer concerned about the intervention curriculum. The research protocol was approved by the University of Massachusetts Medical School investigation committee and the institutional review board of the Bahamian Princess Margaret Hospital, Public Hospitals Authority.

## Discussion

Implementation of evidence-based prevention programs in school settings remains low; sustained implementation is even lower. To address the important challenges confronting worldwide implementation of evidence-based programs in school settings, this research is designed to identify which structures effectively support high-level implementation fidelity and sustainability of prevention programs. This ongoing study explores several theory-driven implementation strategies to increase sustained teacher implementation fidelity to increase the public health impact of evidence-based interventions. The Bahamas has identified FOYC+CImPACT as one of the core evidence-based components of its UNAIDS Fast Track strategy to eliminate the global AIDS epidemic by 2030 and is committed to a data-based implementation plan to improve intervention delivery and maximize the program’s impact among Bahamian students nationwide. The commitment and ongoing involvement of both the MOE and MOH will allow this nationwide research to serve as a global model for the UNAIDS Fast Track strategy. This research program has potential to make significant contributions to advancing school-based HIV prevention research and implementation science.

## Appendix

Multimedia Appendix 1 Peer review reports.

## Abbreviations

ANOVA: analysis of variance
BMF: biweekly monitoring and feedback
CImPACT: Caribbean Informed Parents and Children Together
FOYC: Focus on Youth in the Caribbean
HFLE: Health and Family Life Education
MOE: Ministry of Education
MOH: Ministry of Health
MOST: multiphase optimization strategy
NICHD: Eunice Kennedy Shriver National Institute of Child Health and Human Development
PrEP: preexposure prophylaxis
SAM: site-based assistance and mentorship
UN: United Nations
UNAIDS: Joint United Nations Programme on HIV/AIDS

## References

[1] (2014). Fast track: ending the HIV epidemic by 2030. Joint United Nations Programme on HIV/AIDS.

[2] Porth T, Suzuki C, Gillespie A (2014). Disparities and trends in AIDS mortality among adolescents living with HIV in low- and middle-income countries.

[3] Children and AIDS (2014). Children, Adolescents and AIDS. Digital Release, November 2014.

[4] (2011). Political declaration on HIV and AIDS: intensifying our efforts to eliminate HIV/AIDS. United Nations General Assembly.

[5] The Millenium Development Goals Report, 2014. United Nations Development Programme.

[6] Vermund SH, Tique JA, Cassell HM, Pask ME, Ciampa PJ, Audet CM (2013). Translation of biomedical prevention strategies for HIV: prospects and pitfalls. J Acquir Immune Defic Syndr.

[7] Padian NS, McCoy SI, Karim SSA, Hasen N, Kim J, Bartos M, Katabira E, Bertozzi SM, Schwartländer B, Cohen MS (2011). HIV prevention transformed: the new prevention research agenda. Lancet.

[8] Chang LW, Serwadda D, Quinn TC, Wawer MJ, Gray RH, Reynolds SJ (2013). Combination implementation for HIV prevention: moving from clinical trial evidence to population-level effects. Lancet Infect Dis.

[9] Adelman HS, Taylor L (2003). On sustainability of project innovations as systemic change. J Educ Psychol Consult.

[10] Foy R, Sales A, Wensing M, Aarons GA, Flottorp S, Kent B, Michie S, O'Connor D, Rogers A, Sevdalis N, Straus S, Wilson P (2015). Implementation science: a reappraisal of our journal mission and scope. Implement Sci.

[11] Lyon AR, Frazier SL, Mehta T, Atkins MS, Weisbach J (2011). Easier said than done: intervention sustainability in an urban after-school program. Adm Policy Ment Health.

[12] Dusenbury L, Brannigan R, Falco M, Hansen W (2003). A review of research on fidelity of implementation: implications for drug abuse prevention in school settings. Health Educ Res.

[13] McIntosh K, Mercer S, Nese R, Strickland-Cohen M, Kittelman A, Hoselton R, Horner R (2018). Factors predicting sustained implementation of a universal behavior support framework. Educ Res.

[14] Wang B, Deveaux L, Knowles V, Koci V, Rolle G, Lunn S, Li X, Stanton B (2015). Fidelity of implementation of an evidence-based HIV prevention program among Bahamian sixth grade students. Prev Sci.

[15] Bellg AJ, Borrelli B, Resnick B, Hecht J, Minicucci DS, Ory M, Ogedegbe G, Orwig D, Ernst D, Czajkowski S, Treatment Fidelity Workgroup of the NIH Behavior Change Consortium (2004). Enhancing treatment fidelity in health behavior change studies: best practices and recommendations from the NIH Behavior Change Consortium. Health Psychol.

[16] Rohrbach L, Ringwalt C, Ennett S, Vincus A (2005). Factors associated with adoption of evidence-based substance use prevention curricula in US school districts. Health Educ Res.

[17] Veniegas RC, Kao UH, Rosales R, Arellanes M (2009). HIV prevention technology transfer: challenges and strategies in the real world. Am J Public Health.

[18] (2014). The importance of contextual fit when implementing evidence-based interventions. Office of the Assistant Secretary for Planning and Evaluation, HHS.

[19] Piot P, Bartos M, Larson H, Zewdie D, Mane P (2008). Coming to terms with complexity: a call to action for HIV prevention. Lancet.

[20] Forman SG, Shapiro ES, Codding RS, Gonzales JE, Reddy LA, Rosenfield SA, Sanetti LMH, Stoiber KC (2013). Implementation science and school psychology. Sch Psychol Q.

[21] Aarons GA, Hurlburt M, Horwitz SM (2011). Advancing a conceptual model of evidence-based practice implementation in public service sectors. Adm Policy Ment Health.

[22] Little MA, Sussman S, Sun P, Rohrbach LA (2013). The effects of implementation fidelity in the towards no drug abuse dissemination trial. Health Educ (Lond).

[23] Kershner S, Flynn S, Prince M, Potter SC, Craft L, Alton F (2014). Using data to improve fidelity when implementing evidence-based programs. J Adolesc Health.

[24] Forman SG, Barakat NM (2011). Cognitive-behavioral therapy in the schools: bringing research to practice through effective implementation. Psychol Schs.

[25] Wang B, Stanton B, Deveaux L, Poitier M, Lunn S, Koci V, Adderley R, Kaljee L, Marshall S, Li X, Rolle G (2015). Factors influencing implementation dose and fidelity thereof and related student outcomes of an evidence-based national HIV prevention program. Implementation Sci.

[26] Stanton B (2015). Teachers' patterns of implementation of an evidence-based intervention and their impact on student outcomes: results from a nationwide dissemination over 24-months follow-up. AIDS Behav.

[27] Forman SG, Olin SS, Hoagwood KE, Crowe M, Saka N (2008). Evidence-based interventions in schools: developers’ views of implementation barriers and facilitators. School Mental Health.

[28] Wenger E (1998). Communities of Practice: Learning, Meaning, and Identity.

[29] Li LC, Grimshaw JM, Nielsen C, Judd M, Coyte PC, Graham ID (2009). Evolution of Wenger's concept of community of practice. Implement Sci.

[30] Norman CD, Huerta T (2006). Knowledge transfer & exchange through social networks: building foundations for a community of practice within tobacco control. Implement Sci.

[31] Feldman M, Silapaswan A, Schaefer N, Schermele D (2014). Is there life after DEBI? Examining health behavior maintenance in the diffusion of effective behavioral interventions initiative. Am J Community Psychol.

[32] Deveaux L, Lunn S, Bain RM, Gomez P, Kelly T, Brathwaite N, Russell-Rolle G, Li X, Stanton B (2011). Focus on youth in the Caribbean: beyond the numbers. J Int Assoc Physicians AIDS Care (Chic).

[33] Global AIDS update 2018: miles to go: the response to HIV in the Caribbean.

[34] HIV/AIDS surveillance fact sheet. Bahamas Ministry of Health.

[35] Wang B, Stanton B, Knowles V, Russell-Rolle G, Deveaux L, Dinaj-Koci V, Li X, Brathwaite N, Lunn S (2014). Sustained institutional effects of an evidence-based HIV prevention intervention. Prev Sci.

[36] Aarons GA, Green AE, Trott E, Willging CE, Torres EM, Ehrhart MG, Roesch SC (2016). The roles of system and organizational leadership in system-wide evidence-based intervention sustainment: a mixed-method study. Adm Policy Ment Health.

[37] Drake PM, Firpo-Triplett R, Glassman JR, Ong SL, Unti L (2015). A randomized-controlled trial of the effects of online training on implementation fidelity. Am J Sex Educ.

[38] Wang B, Stanton B, Lunn S, Patel P, Koci V, Deveaux L (2017). Development of a brief pre-implementation screening tool to identify teachers who are at risk for not implementing intervention curriculum and high-implementing teachers. Health Educ Behav.

[39] Friedman I (1999). Teacher-perceived work autonomy: the concept and its measurement. Educ Psychol Meas.

[40] Schutte L, Meertens RM, Mevissen FEF, Schaalma H, Meijer S, Kok G (2014). Long live love. The implementation of a school-based sex-education program in The Netherlands. Health Educ Res.

[41] Martínez J, Vicario-Molina I, González E, Ilabaca P (2014). Sex education in Spain: the relevance of teachers’ training and attitudes / Educación sexual en España: importancia de la formación y las actitudes del profesorado. Infancia y Aprendizaje.

[42] Collins LM, Baker TB, Mermelstein RJ, Piper ME, Jorenby DE, Smith SS, Christiansen BA, Schlam TR, Cook JW, Fiore MC (2011). The multiphase optimization strategy for engineering effective tobacco use interventions. Ann Behav Med.

[43] Collins LM, Dziak JJ, Li R (2009). Design of experiments with multiple independent variables: a resource management perspective on complete and reduced factorial designs. Psychol Methods.

[44] Collins LM, Kugler KC, Gwadz MV (2016). Optimization of multicomponent behavioral and biobehavioral interventions for the prevention and treatment of HIV/AIDS. AIDS Behav.

[45] Wang B, Stanton B, Deveaux L, Lunn S, Rolle G, Adderley R, Poitier M, Koci V, Marshall S, Gomez P (2017). Multi-year school-based implementation and student outcomes of an evidence-based risk reduction intervention. Implement Sci.

[46] Han SS, Weiss B (2005). Sustainability of teacher implementation of school-based mental health programs. J Abnorm Child Psychol.

[47] Byrne B (2013). Structural Equation Modeling with LISREL, PRELIS, and SIMPLIS: Basic Concepts, Applications, and Programming.

[48] Hu L, Bentler PM (1999). Cutoff criteria for fit indexes in covariance structure analysis: conventional criteria versus new alternatives. Struct Equ Modeling.

