# Supplementary material for: Bahamas National Implementation Project: Proposal for Sustainability of an Evidence-based HIV Prevention Intervention in a School Setting
Source: JMIR Res Protoc. 2020 Aug 21;9(8):e14816. doi: 10.2196/14816 (PMC7474416; doi:10.2196/14816)
Supplement: Multimedia Appendix 1 [file resprot_v9i8e14816_app1.pdf]

**SUMMARY STATEMENT**
**( Privileged Communication )**

**PROGRAM CONTACT:**
**Sonia Lee**
**(301)594-4783**
**leesonia@mail.nih.gov**

***Release Date:*** **11/10/2017**
***Revised Date:***

***Application Number:*** **1 R01 HD095765-01**

**Principal Investigators (Listed Alphabetically):**

**STANTON, BONITA FRANCES**
**WANG, BO (Contact)**

**Applicant Organization: UNIV OF MASSACHUSETTS MED SCH WORCESTER**

***Review Group:*** **DIRH**
**Dissemination and Implementation Research in Health Study Section**
**AIDS**

***Meeting Date:*** **10/18/2017**
***Council:*** **JAN 2018**
***Requested Start:*** **04/01/2018**
***RFA/PA:*** **PAR16-238**
***PCC:*** **MPIDB-SL**
***Dual IC(s):*** **MH, MD**

***Project Title:*** **National Implementation of FOYC+CImPACT in the Bahamas: implementation strategies and improved outcomes.**
***SRG Action:*** **Impact Score:25 Percentile:10**
***Next Steps:*** **Visit https://grants.nih.gov/grants/next_steps.htm**
**Human Subjects: 30-Human subjects involved - Certified, no SRG concerns**
**Animal Subjects: 10-No live vertebrate animals involved for competing appl.**
**Gender: 1A-Both genders, scientifically acceptable**
**Minority: 5A-Only foreign subjects, scientifically acceptable**
**Children: 1A-Both Children and Adults, scientifically acceptable**
**Clinical Research - not NIH-defined Phase III Trial**

| Project Year | Direct Costs Requested | Estimated Total Cost |
|---|---|---|
| 1 | 499,966 | 665,742 |
| 2 | 499,709 | 665,400 |
| 3 | 499,808 | 665,532 |
| 4 | 499,875 | 665,621 |
| 5 | 499,825 | 665,554 |
| TOTAL | 2,499,183 | 3,327,849 |

**ADMINISTRATIVE BUDGET NOTE: The budget shown is the requested budget and has not been adjusted to reflect any recommendations made by reviewers. If an award is planned, the costs will be calculated by Institute grants management staff based on the recommendations outlined below in the COMMITTEE BUDGET RECOMMENDATIONS section.**

**1R01HD095765-01 Wang, Bo**

**RESUME AND SUMMARY OF DISCUSSION:** This application proposes to monitor and evaluate the implementation fidelity of two evidence-based HIV risk reduction programs, Focus on Youth in the Caribbean (FOYC) and Caribbean Informed Parents and Children Together (CImPACT), as they are implemented, nationally, by 6$^{th}$ grade teachers in the Bahamas. If successful, the study results will support the national sustainability, implementation fidelity and maintenance the effectiveness of the FOYC and CImPACT evidence-based HIV-prevention programs. The panel agreed that the study was highly significant and the scientific premise was sound as evidenced by the literature citing the increasing global impact of HIV on adolescents and preliminary studies demonstrating the effectiveness of the FOYC and CImPACT interventions, when delivered by high-performing teachers, on increasing students' HIV-related knowledge, condom-use skills, self-efficacy and self-reported risk behaviors. Reviewers viewed the idea of integrating a Multiphase Optimization StraTegy design and incrementally expanding the investigative team's national trial, as moderately innovative. This is an outstanding multidisciplinary team with complementary expertise, a long collaborative history and a strong multi-principal investigator plan with a supportive environment to achieve the study's aims. The panel identified additional strengths in the approach, including: the development and testing of fidelity maintenance; use of the Fidelity through Technical Assistance and Training implementation approach; and a strong analytic plan, strengthening the scientific rigor. Reviewers also noted weaknesses, including: lack of detail regarding the sustainability of teacher reimbursement; no clarity about incentives for team leaders to serve in a site-based assistance/mentorship role; and insufficient details about the sustainability of the inclusion of the CImPACT component of the proposed study. The committee viewed these weaknesses as addressable and not detracting from application's high merit. Overall, the panel expressed high enthusiasm for the application and agreed that the findings are expected to have a high impact on advancing implementation science and delivering an evidence-based HIV prevention curriculum for students enrolled in grades 6 to 9, and their parents, in the Bahamas.

**DESCRIPTION (provided by applicant):** Over the past two decades, the Bahamian Ministries of Education (MOE) and Health (MOH) and our research team have adapted a CDC "Best Evidence" HIV Prevention Program to produce the "Focus on Youth in the Caribbean (FOYC) and Caribbean Informed Parents and Children Together" (CImPACT) risk reduction program to address the HIV epidemic in The Bahamas. Two randomized, controlled longitudinal trials of FOYC and FOYC+CImPACT found the programs to be effective in improving knowledge, condom-use skills and/or self-reported risk behaviors. In 2010 the MOE decided to include FOYC (logistically less complex than FOYC+CImPACT) in the government grade-6 curriculum nationwide, with boosters in grades-7 and -8. The MOE now plans to expand the offering to the more effective but logistically more complex FOYC+ CImPACT version. The MOE, MOH and our research team examined factors associated with fidelity of teaching FOYC among 283 teachers, and the relationship between fidelity of teaching with student outcomes among 4,411 students. Teachers taught slightly over 50% of FOYC's core activities. Evaluations indicate that students taught by "low-performing" teachers exhibited poorer outcomes in HIV/AIDS knowledge, condom-use skills, self- efficacy, and intentions; the reverse was true for students of "high-performing" teachers. Screening instruments and tracking processes to identify teachers at risk of low implementation, and evidence-based implementation support and remediation programs have been identified/developed for use throughout implementation. Biweekly "real-time" data-gathering and analysis to inform ongoing decision-making about adjustments or changes to be made to the implementation plan of FOYC+CImPACT by the MOE and MOH are the focus of this proposal. A Bahamian-specific adaptation of communities of practice (CoP), implementation monitoring and feedback, and innovative training are strategies to enhance teachers' implementation. The proposed research addresses three hypotheses of public health importance: 1. A more intensive training and supervision program for at-risk or moderate-performing teachers will enhance their implementation fidelity to the average level of the high-performing group (85%); 2. An HIV-prevention program delivered at the national level can be a) implemented with fidelity (delivering >85% of core activities in

both FOYC and CImPACT consistent with that described by the developers) in grade-6; and, b) sustained over time (monitored annually); and, 3. Student outcomes (knowledge, reproductive health skills, perceptions and self-reported behaviors) will: a) continue to be highly correlated with implementation fidelity; and, b) be sustained over time (assessed annually through grade 9). We aim to determine if the provision of either or both a) biweekly monitoring, feedback and support through a "community of practice" to at-risk and moderate-performing teachers; and b) an enhanced decision-making platform by the MOE and MOH based on the real-time implementation data will: 1) increase national implementation fidelity; and 2) result in sustained implementation over time.

**PUBLIC HEALTH RELEVANCE:** The global public health impact of the HIV/AIDS epidemic is unparalleled in the last half-century. Currently the global public health community is at a critical juncture, working towards the possibility of eradicating the global AIDS epidemic by the year 2030. However, this excitement is tempered by mounting concern that if this goal is not achieved, emerging resistant HIV strains and declining resources targeting the problem may result in a resurgence of the epidemic. The proposed research will support the efforts of the entire nation of The Commonwealth of The Bahamas and serve as a prototype for other nations in the quest for elimination of the HIV/AIDS epidemic by 2030. It employs a theory-based, practical, data-driven implementation process to deliver an evidence-based HIV prevention curriculum through the government school system to all students throughout the nation enrolled in grades-6 to 9 and their parents.

**CRITIQUE 1**

Significance: 3
Investigator(s): 1
Innovation: 1
Approach: 5
Environment: 2

**Overall Impact:** The focus of this application is to limit HIV infections by increasing adherence to an existing sexual health intervention for junior high youth, taught by teachers, in the Bahamas. The research team has a long history of collaboration with the Bahamian Ministries of Health and Education on HIV education with youth. The applicants utilize a multiphase optimization strategy (MOST) to inform their implementation approach and will utilize a full factorial design to assess the best implementation monitoring system to improve fidelity and sustainable implementation across time. The intervention has 2 components. The first is Focus on Youth Caribbean, (FOYC) which is a classroom based intervention consisting of 8 sessions occurring in the 6$^{th}$ grade (with subsequent booster sessions in the 7$^{th}$ and 8$^{th}$ grades). The second is Caribbean Informed Parents and Children Together (CImPACT) that includes a single session with parents and youth to address communication and a condom demonstration. Previous research of FOYC + CImPACT by this group with Bahamian youth demonstrated effectiveness in attitude change and rates of condom use. A subsequent R01 focused on the national implementation of FOYC alone. Significant variation in dosage and curricula fidelity were noted. Drs. Wang and Stanton are co-PIs for this project. Dr. Wang is an associate professor and has a strong publication history in teen sexual health as well as the development and evaluation sexual education programs. Dr. Stanton, a professor and Dean, has mentored Dr. Wang for several years; they have collaborated extensively and have a clear multiple PI leadership plan. Additional strengths of the current application include using the Exploration, Preparation, Implementation, Sustainment (EPIS) model to guide the implementation process. Score driving weaknesses include minimal discussion of school leadership support, the new inclusion of CImPACT in this national implementation study, and issues regarding the site-based assistance/mentorship condition.

**1. Significance:**

**Strengths**

- Focusing on partnering with teachers to implement the HIV risk reduction curricula is a significant need that warrants attention in the implementation research literature. Thus, there is a strong scientific premise for this project.

**Weaknesses**

- Minor: Evidence of sustainability is offered as pilot data. However, sustainability was described as improved student outcomes when teachers received additional FOYC training and that teachers who received training continued to teach elements of it after being placed in other schools. Thus, the pilot data related to sustainability is not relevant to the proposal.
- Moderate: School leadership organizational support is given short-shrift in the application. School level factors (principal support) are listed in Table 1 as an issue impacted implementation and in Figure 2 in the SEM model. However, principal and school leadership support is not described further in the application nor is recruitment of principals described in the human subjects section.

**2. Investigator(s):**

**Strengths**

- Drs. Wang and Stanton are productive investigators. They have collaborated successfully on previous projects with the same population.
- The collaborators in the research team bring additional research and clinical expertise in multiple areas.
- The extended research team also has strong publication histories in the relevant research literature specific to this project.

**Weaknesses**

- None noted.

**3. Innovation:**

**Strengths**

- A Type 3 hybrid design will be used on a national scale to improve fidelity to an effective HIV reduction intervention with early adolescents.

**Weaknesses**

- None noted.

**4. Approach:**

**Strengths**

- The applicants have very strong pilot data that buttresses their current analysis plan. For instance, they are tested and validated a teacher fidelity scale that will identify teachers that need additional intervention to improve fidelity.
- A number of implementation fidelity measures will be included. For instance, a significant proportion of FOYC (10%) and CImPACT (20%) will be independently monitored.

**Weaknesses**

- Minor: A significant barrier to implementing CImPACT was teacher reimbursement for time to administer the intervention. As such, teachers will be reimbursed for conducting CImPACT with

families. It is unclear if this is a sustainable model for the Bahamian Ministries of Health or Education.

- Minor: In the previous expansion of FOYC across the nation, the Bahamian Ministry of Education conducted teacher workshops. Only 49% of teachers attended and 11% had attended a workshop in the original FOYC trial. As such, information regarding training will be placed on a flash drive for teachers. Nonetheless, it will not be possible to track utilization of the flash drive and only attendance at workshops can be measured by the applicants.
- Moderate: As a part of the site-based assistance/mentorship (SAM) condition of the full factorial design, schools will be randomized into 3 conditions (no SAM, SAM, and enhance SAM). High-performing teachers, FOYC + CImPACT guidance counselors and retired HFLE teachers will serve as team leaders. For SAM sites, team leaders will meet weekly with 6th grade teachers and HFLE teachers to discuss progress. Team leaders for enhanced SAM will observe low performing teachers to improve fidelity. These are significant activities that are core aspects of the study. It does not appear that team leaders are incentivized for this additional work that is quite time-consuming.
- Moderate: In the previous national expansion of FOYC, the CImPACT portion of the intervention was not included as it was about a priority for the Bahamian Ministry of Education. In the current application, CImPACT is again included as a core feature of FOYC. This is a significant amendment to the previous national expansion that will warrant an additional focus for sustainability. Given that all decisions for implementation of FOYC + CImPACT will be determined by the Bahamian government agencies, the shifting focus is significant.

**5. Environment:**

**Strengths**

- A research team has an exceptionally strong history of collaboration with Bahamian agencies, teachers and students.

**Weaknesses**

- Minor: Dr. Stanton is a recent founding Dean of a medical school and has several research projects that continue, including a center grant. She is devoting a relatively small portion of her time (10%) to this project considering she is a co-PI.

**Protections for Human Subjects:**

Acceptable Risks and/or Adequate Protections

Data and Safety Monitoring Plan (Applicable for Clinical Trials Only):

Acceptable

**Inclusion of Women, Minorities and Children:**

- Sex/Gender: Distribution justified scientifically
- Race/Ethnicity: Distribution justified scientifically
- For NIH-Defined Phase III trials, Plans for valid design and analysis: Not applicable
- Inclusion/Exclusion of Children under 18: Including ages <18; justified scientifically

**Vertebrate Animals:**

Not Applicable (No Vertebrate Animals)

**Biohazards:**

Not Applicable (No Biohazards)

**Applications from Foreign Organizations:**

Justified

**Select Agents:**

Not Applicable (No Select Agents)

**Resource Sharing Plans:**

Acceptable

**Authentication of Key Biological and/or Chemical Resources:**

Not Applicable (No Relevant Resources)

**Budget and Period of Support:**

Recommend as Requested

**CRITIQUE 2**

Significance: 1
Investigator(s): 1
Innovation: 2
Approach: 2
Environment: 1

**Overall Impact:** This new R01 application proposes a five-year study of national implementation of a school-based HIV prevention program in the Bahamas. The study will also investigate the effects of additional training with teachers who do not implement the intervention with high fidelity. The scientific premise is strong based on the argument for the need for enhanced teacher training and support. The environment is excellent, with previous buy-in from the Ministry of Health and Education in the Bahamas, and previous collaboration between the investigators and the Ministries. The investigative team is strong. Innovation is low to moderate. The approach is strong, with a weakness being the lack of attention to how to ensure buy in from low performing teachers. Overall this project could have major impact on public health practice and health outcomes in the Bahamas and provide an example of effective implementation for other countries.

**1. Significance:**

**Strengths**

- Improving the implementation and effectiveness of HIV prevention within Bahamian schools addresses a significant problem.
- The scientific premise is strong based on the argument for the need for enhanced teacher training and support.

**Weaknesses**

- None noted.

**2. Investigator(s):**

**Strengths**

- The investigators appear to have the requisite skills and experience to conduct this five-year R01 project. The extensive experience and prior work on HIV prevention programs in the Bahamas sets the stage for this project.

**Weaknesses**

- None noted.

**3. Innovation:**

**Strengths**

- None noted.

**Weaknesses**

- Minor: The project is not particularly innovative.

**4. Approach:**

**Strengths**

- The approach has many strengths. All aspects of the study are well described.
- The previous studies set the stage for the proposed work.
- The rationale for the project was very well described.
- The implementation aspect of the project is central and very well explained.

**Weaknesses**

- Moderate: The project focuses on improving implementation of the effective HIV risk reduction and sex education programs by identifying and targeting teachers who do not implement as well as they could/should. How will they ensure buy in and participation by low-participation teachers? The incentives for increased participation were not well described. If they cannot ensure these teachers will attempt to improve their performance, then the project will not be successful. However, I suppose that is part of the rationale of the project.

**5. Environment:**

**Strengths**

- The environment at the home institution and in the Bahamas, is strong. The existing strong relationship with the schools is key to this project working well.

**Weaknesses**

- None noted.

**Protections for Human Subjects:**

Acceptable Risks and/or Adequate Protections

- There are minimal risks involved here, as the research involves improving the implementation of an existing school-based intervention.

Data and Safety Monitoring Plan (Applicable for Clinical Trials Only):

Acceptable

**Inclusion of Women, Minorities and Children:**

- Sex/Gender: Distribution justified scientifically
- Race/Ethnicity: Distribution justified scientifically
- For NIH-Defined Phase III trials, Plans for valid design and analysis: Not applicable
- Inclusion/Exclusion of Children under 18: Excluding ages <18; justified scientifically
- Teachers who deliver the intervention are the participants in the project

**Vertebrate Animals:**

Not Applicable (No Vertebrate Animals)

**Biohazards:**

Not Applicable (No Biohazards)

**Applications from Foreign Organizations:**

Not Applicable (No Foreign Organizations)

**Select Agents:**

Not Applicable (No Select Agents)

**Resource Sharing Plans:**

Not Applicable (No Relevant Resources)

**Authentication of Key Biological and/or Chemical Resources:**

Not Applicable (No Relevant Resources)

**Budget and Period of Support:**

Recommend as Requested

Recommended budget modifications or possible overlap identified:

- This is a lot packed into a 5-year project, but the investigators have had success in conducting similar large-scale work.

**CRITIQUE 3**

Significance: 1
Investigator(s): 1
Innovation: 3
Approach: 2
Environment: 1

**Overall Impact:** The proposed study will examine the effect of two enhanced teacher training protocols (peer mentorship and an enhanced decision-making platform) on implementation fidelity and sustained implementation of an HIV/AIDS preventive intervention implemented nationally in the Bahamas. The scientific premise of the study is strong. Multiple rigorous trials demonstrate the effectiveness of Focus on Youth Caribbean (FOYC) and Caribbean Informed Parents and Children Together (CImPACT) on improved HIV/AIDS knowledge, condom use and self-efficacy among 6$^{th}$ grade youth in the Bahamas. Youth outcomes were directly linked with the fidelity of curriculum implementation with students taught by high performing teachers demonstrating improved outcomes. Additional research showed that training in interactive teaching, completion of the FOYC training and comfort implementing the curriculum predicted higher implementation fidelity. The proposed study will build on these results by testing two teaching training protocols that directly target predictors of implementation fidelity previously identified. The study has a number of noteworthy strengths including its integration into a national implementation study, a strong partnership with the Ministries of Education and Health in the Bahamas, impressive preliminary research, and the use of MOST trial to identify an optimal package of training enhancements. Weaknesses included a moderate expansion in scope from the prior trial and the failure to include new HIV/AIDS cases as an indicator of program impact. Overall, the strengths outweigh the weaknesses and enthusiasm is moderate to high for this application.

**1. Significance:**

**Strengths**

- The proposed study has the potential to reduce the risk of HIV/AIDS in emerging adults (< 25 years), the most vulnerable age group for new HIV/AIDS infections.
- If successful, the proposed study will identify scalable implementation strategies to support sustained implementation of evidence-based interventions.
- The proposed study has the potential to improve regional and national implementation of EBIs and subsequently their public health impact.

**Weaknesses**

- None noted.

**2. Investigator(s):**

**Strengths**

- Co-PIs Wang and Stanton have published 43 papers together and have collaboratively implemented multiple studies in the Bahamas.
- The multi-PI plan clearly specifies the responsibilities of each PI.
- PI Wang has a background in implementation science, HIV/AIDS prevention and adolescent risk behaviors and has collaborated on two prior studies in the Bahamas.
- PI Stanton has over 25 years of experience in HIV prevention.
- Co-I Ghosh is a biostatistician and has experience utilizing the MOST design.
- The investigative team is highly qualified to implement the proposed study.

**Weaknesses**

- None noted.

**3. Innovation:**

**Strengths**

- The proposed study is the first national implementation study to integrate multiple strategies to promote implementation and sustainability.
- The integration of a MOST design is novel and permits identification of the optimal package of teacher support strategies.

**Weaknesses**

- Moderate: The proposed study moderately expands the prior national trial (a careful next step rather than an innovative leap).

**4. Approach:**

**Strengths**

- An evidence-based intervention.
- The proposed study is built on an impressive series of preliminary studies.
- The Fidelity through Informed Technical Assistance and Training (FITT) implementation approach is utilized.
- Multiple measures (e.g., Teachers Implementation Checklist, X) were developed and validated by the research team.
- Table 1 effectively summarizes differences between the previous national trial and the proposed study. In particular, the proposed study will develop and test fidelity maintenance strategies not considered in the original trial.
- The proposed study draws on national data compiled by the Ministry of Education in the Bahamas.
- The analytic plan clearly maps on to the research questions and communicates the relevance and implications of each strategy.
- The hypothesized model outlined in Figure 2 is conceptually and empirically robust.

**Weaknesses**

- Moderate: Examining changes in new HIV/AIDS infections in the target age group would provide a clear indication of the public health impact of the program.

**5. Environment:**

**Strengths**

- The research team has a long standing successful collaboration with the Bahamas Ministry of Education and Ministry of Health.
- The University of Massachusetts Medicine School, Seton Hall University and University of South Carolina have the necessary research infrastructure to support successful implementation of the proposed study.

**Weaknesses**

- None noted.

**Protections for Human Subjects:**

Acceptable Risks and/or Adequate Protections

- Appropriate steps are taken to minimize participant risk.

Data and Safety Monitoring Plan (Applicable for Clinical Trials Only):

Acceptable

**Inclusion of Women, Minorities and Children:**

- Sex/Gender: Distribution justified scientifically
- Race/Ethnicity: Distribution justified scientifically
- For NIH-Defined Phase III trials, Plans for valid design and analysis: Scientifically acceptable
- Inclusion/Exclusion of Children under 18: Excluding ages <18; justified scientifically

**Vertebrate Animals:**

Not Applicable (No Vertebrate Animals)

**Biohazards:**

Not Applicable (No Biohazards)

**Applications from Foreign Organizations:**

Not Applicable (No Foreign Organizations)

**Select Agents:**

Not Applicable (No Select Agents)

**Resource Sharing Plans:**

Acceptable

**Authentication of Key Biological and/or Chemical Resources:**

Not Applicable (No Relevant Resources)

**Budget and Period of Support:**

Recommend as Requested

**THE FOLLOWING SECTIONS WERE PREPARED BY THE SCIENTIFIC REVIEW OFFICER TO SUMMARIZE THE OUTCOME OF DISCUSSIONS OF THE REVIEW COMMITTEE, OR REVIEWERS' WRITTEN CRITIQUES, ON THE FOLLOWING ISSUES:**

**PROTECTION OF HUMAN SUBJECTS:** ACCEPTABLE

**INCLUSION OF WOMEN PLAN:** ACCEPTABLE

**INCLUSION OF MINORITIES PLAN:** ACCEPTABLE

**INCLUSION OF CHILDREN PLAN:** ACCEPTABLE

**COMMITTEE BUDGET RECOMMENDATIONS:** The budget was recommended as requested.

---

Footnotes for 1 R01 HD095765-01; PI Name: Wang, Bo

NIH has modified its policy regarding the receipt of resubmissions (amended applications). See Guide Notice NOT-OD-14-074 at http://grants.nih.gov/grants/guide/notice-files/NOT-OD-14-074.html. The impact/priority score is calculated after discussion of an application by averaging the overall scores (1-9) given by all voting reviewers on the committee and multiplying by 10. The criterion scores are submitted prior to the meeting by the individual reviewers assigned to an application, and are not discussed specifically at the review meeting or calculated into the overall impact score. Some applications also receive a percentile ranking. For details on the review process, see http://grants.nih.gov/grants/peer_review_process.htm#scoring.

MEETING ROSTER

Dissemination and Implementation Research in Health Study Section
Healthcare Delivery and Methodologies Integrated Review Group
CENTER FOR SCIENTIFIC REVIEW
DIRH
10/18/2017 - 10/19/2017

Notice of NIH Policy to All Applicants: Meeting rosters are provided for information purposes only. Applicant investigators and institutional officials must not communicate directly with study section members about an application before or after the review. Failure to observe this policy will create a serious breach of integrity in the peer review process, and may lead to actions outlined in NOT-OD-14-073 at https://grants.nih.gov/grants/guide/notice-files/NOT-OD-14-073.html and NOT-OD-15-106 at https://grants.nih.gov/grants/guide/notice-files/NOT-OD-15-106.html, including removal of the application from immediate review.

CHAIRPERSON(S)

SIMON, MELISSA ANDREA, MD, MPH
PROFESSOR AND VICE CHAIR
DEPARTMENT OF OBSTETRICS AND GYNECOLOGY
AND PREVENTIVE MEDICINE
FEINBERG SCHOOL OF MEDICINE
NORTHWESTERN UNIVERSITY
CHICAGO, IL 60611

MEMBERS

AALSMA, MATTHEW, PHD
PROFESSOR
SECTION OF ADOLESCENT MEDICINE
INDIANA UNIVERSITY SCHOOL OF MEDICINE
INDIANAPOLIS, IN 46203

BARTELS, STEPHEN, MD
PROFESSOR/DIRECTOR OF DARTMOUTH
CENTERS FOR HEALTH AND AGING
DEPARTMENTS OF PSYCHIATRY
COMMUNITY AND FAMILY MEDICINE AND HEALTH POLICY
DARTMOUTH MEDICAL SCHOOL
LEBANON, NH 03766

BEETS, MICHAEL W, PHD *
ASSOCIATE PROFESSOR
DEPARTMENT OF EXERCISE SCIENCE
ARNOLD SCHOOL OF PUBLIC HEALTH
UNIVERSITY OF SOUTH CAROLINA
COLUMBIA, SC 29208

BOYNTON-JARRETT, RENEE DANIELLE, MD, SCD *
ASSOCIATE PROFESSOR
SCHOOL OF MEDICINE
BOSTON UNIVERSITY
BOSTON, MA 02118

CARRASQUILLO, OLVEEN, MD, MPH
PROFESSOR OF MEDICINE AND PUBLIC HEALTH SCIENCES
CHIEF, DIVISION OF INTERNAL MEDICINE GERIATRICS
MILLER SCHOOL OF MEDICINE
UNIVERSITY OF MIAMI
MIAMI, FL 33101

CATLEY, DELWYN, PHD *
PROFESSOR
DEPARTMENT OF PEDIATRICS
CHILDREN'S MERCY HOSPITAL
KANSAS CITY, MO 64108

CHEN, JIE, PHD *
ASSOCIATE PROFESSOR
DEPARTMENT OF HEALTH SERVICES ADMINISTRATION
SCHOOL OF PUBLIC HEALTH
UNIVERSITY OF MARYLAND
COLLEGE PARK, MD 20742

CORSO, PHAEDRA S, PHD *
PROFESSOR
DEPARTMENT OF HEALTH POLICY
AND MANAGEMENT
UNIVERSITY OF GEORGIA
ATHENS, GA 30602

DAVISON, KIRSTEN, PHD *
ASSOCIATE PROFESSOR
DEPARTMENT OF NUTRITION
HARVARD SCHOOL OF PUBLIC HEALTH
BOSTON, MA 02115

DUBBERT, PATRICIA M, PHD *
PROFESSOR (RETIRED)
UNIVERSITY OF ARKANSAS FOR MEDICAL SCIENCES
LITTLE ROCK, AR 72205

ELLERBECK, EDWARD F, MD, MPH
PROFESSOR
DEPARTMENT OF PREVENTIVE MEDICINE
UNIVERSITY OF KANSAS MEDICAL CENTER
KANSAS CITY, KS 66160-7313

FAN, VINCENT S, MD, MPH *
ASSOCIATE PROFESSOR
VA PUGET SOUND HEALTH CARE SYSTEM
SEATTLE, WA 98101

FRITZ, JULIE M, PHD
PROFESSOR
ASSOCIATE DEAN FOR RESEARCH
COLLEGE OF HEALTH
UNIVERSITY OF UTAH
SALT LAKE CITY, UT 84102

GAJIC, OGNJEN, MD *
PROFESSOR
DEPARTMENT OF MEDICINE
COLLEGE OF MEDICINE
MAYO CLINIC, ROCHESTER
ROCHESTER, MN 55905

HANNON, MARGARET, PHD
ASSOCIATE PROFESSOR
DEPARTMENT OF HEALTH SERVICES
UNIVERSITY OF WASHINGTON
SEATTLE, WA 98105

HARE, MARION, MD *
ASSOCIATE PROFESSOR
DEPARTMENT OF PREVENTIVE MEDICINE AND PEDIATRICS
UNIVERSITY OF TENNESSEE HEALTH SCIENCE CENTER
MEMPHIS, TN 38163

HOFFMANN, CHRISTOPHER J, MD *
ASSOCIATE PROFESSOR OF MEDICINE
DEPARTMENT OF MEDICINE
DIVISION OF INFECTIOUS DISEASES
JOHNS HOPKINS SCHOOL OF MEDICINE
BALTIMORE, MD 21207

HOUSTON, THOMAS K II, MD, MPH
PROFESSOR AND DIVSION CHIEF
DEPARTMENT OF QUANTITATIVE HEALTH SCIENCES
DIVISION OF HEALTH INFORMATICS AND
IMPLEMENTATION SCIENCE; SCHOOL OF MEDICINE
UNIVERSITY OF MASSACHUSETTS
WORCESTER, MA 01655

HUDSON, SHAWNA V, PHD
PROFESSOR AND  RESEARCH DIVISION CHIEF
DEPARTMENT OF FAMILY MEDICINE
AND COMMUNITY HEALTH
ROBERT WOOD JOHNSON MEDICAL SCHOOL
RUTGERS UNIVERSITY
NEW BRUNSWICK, NJ 08901

HUMPHRIES, MISTY DAWN, MD *
ASSISTANT PROFESSOR
DEPARTMENT OF SURGERY
UC DAVIS HEALTH
SACRAMENTO, CA 95817

JENERETTE, CORETTA M, PHD, RN *
ASSOCIATE PROFESSOR
PHD DIVISION
SCHOOL OF NURSING
UNIVERSITY OF NORTH CAROLINA AT CHAPEL HILL
CHAPEL HILL, NC 27599

JENSEN-DOSS, AMANDA, PHD *
ASSOCIATE PROFESSOR
DEPARTMENT OF PSYCHOLOGY
COLLEGE OF ARTS AND SCIENCES
UNIVERSITY OF MIAMI
CORAL GABLES, FL 33124

KEISER, PHILIP H, MD *
PROFESSOR, INTERNATIONAL HIV PROGRAM AND CLINIC
DIRECTOR
DEPARTMENT OF INTERNATIONAL HEALTH
INFECTIOUS DISEASE DIVISION
UNIVERSITY OF TEXAS MEDICAL BRANCH AT GALVESTON
GALVESTON, TX 77555

KOSHINSKY, HEATHER, PHD *
CHIEF EXECUTIVE OFFICER
INVESTIGEN, INC
HERCULES, CA 94547

KUKAFKA, RITA, DRPH *
ASSOCIATE PROFESSOR OF BIOINFORMATICS AND
SOCIOMEDICAL SCIENCES
DEPARTMENT OF MEDICAL INFORMATICS
COLUMBIA UNIVERSITY
NEW YORK, NY 10032

MAXWELL, ANNETTE, DRPH *
PROFESSOR
DIVISION OF CANCER PREVENTION AND CONTROL
RESEARCH
UNIVERSITY OF  CALIFORNIA, LOS ANGELES
LOS ANGELES, CA 90095-6900

MCLEOD, BRYCE DOUGLAS, PHD
ASSOCIATE PROFESSOR
DEPARTMENT OF PSYCHOLOGY
VIRGINIA COMMONWEALTH UNIVERSITY
RICHMOND, VA 23284

NAYAR, PREETHY, MBBS, PHD *
ASSOCIATE PROFESSOR
COLLEGE OF PUBLIC HEALTH
UNIVERSITY OF NEBRASKA MEDICAL CENTER
OMAHA, NE 68198

NEMETH, LYNNE S, PHD *
PROFESSOR
COLLEGE OF NURSING
MEDICAL UNIVERSITY OF SOUTH CAROLINA
CHARLESTON, SC 29425

POLLINI, ROBIN A, PHD *
ASSOCIATE PROFESSOR
DEPARTMENT OF BEHAVIORAL MEDICINE AND PSYCHIATRY
SCHOOL OF MEDICINE
WEST VIRGINIA UNIVERSITY
MORGANTOWN, WV 26505

SALDANA, LISA, PHD
SENIOR SCIENTIST
OREGON SOCIAL LEARNING CENTER
EUGENE, OR 97401

SALES, ANNE EVELYN, PHD, RN *
PROFESSOR
DEPARTMENT OF LEARNING HEALTH SCIENCES
SCHOOL OF MEDICINE
UNIVERSITY OF MICHIGAN
ANN ARBOR, MI 48109

SARPONG, DANIEL F, PHD *
PROFESSOR OF BIOSTATISTICS, DIRECTOR AND ENDOWED CHAIR
CENTER FOR MINORITY HEALTH AND HEALTH DISPARITIES RESEARCH AND EDUCATION
COLLEGE OF PHARMACY
XAVIER UNIVERSITY OF LOUISIANA
NEW ORLEANS, LA 70125

SELF-BROWN, SHANNON RENEE, PHD *
PROFESSOR
SCHOOL OF PUBLIC HEALTH
GEORGIA STATE UNIVERSITY
ATLANTA, GA 30302

SHORR, RONALD I, MD *
DIRECTOR, GERIATRIC RESEARCH EDUCATION AND CLINICAL CENTER
MALCOM RANDALL VAMC
RESEARCH PROFESSOR
DEPARTMENT OF EPIDEMIOLOGY
UNIVERSITY OF FLORIDA
GAINESVILLE, FL 32608

SMITH, DAVID H, PHD *
SENIOR INVESTIGATOR
CENTER FOR HEALTH RESEARCH
KAISER FOUNDATION HOSPITALS
PORTLAND, OR 97227-1110

SVENSSON, CRAIG K, PHD, PHMD *
DEAN EMERITUS AND PROFESSOR
COLLEGE OF PHARMACY
PURDUE UNIVERSITY
WEST LAFAYETTE, IN 47907

TODD, CATHERINE SUZANNE, MD, MPH *
SCIENTIST I
REPRODUCTIVE, MATERNAL, NEWBORN,
AND CHILD HEALTH DIVISION
FAMILY HEALTH INTERNATIONAL 360
DURHAM, NC 27713

TROTTER, ROBERT TALBOT, PHD *
REGENTS PROFESSOR AND ASSOCIATE VICE PRESIDENT
HEALTH RESEARCH INITIATIVES
NORTHERN ARIZONA UNIVERSITY
FLAGSTAFF, AZ 86011

WEINHARDT, LANCE S, PHD *
PROFESSOR OF COMMUNITY AND BEHAVIORAL HEALTH PROMOTION
ASSOCIATE DEAN FOR RESEARCH
JOSEPH J ZILBER SCHOOL OF PUBLIC HEALTH
UNIVERSITY OF WISCONSIN, MILWAUKEE
MILWAUKEE, WI 53201

WELLS, KRISTEN JENNIFER, PHD *
ASSOCIATE PROFESSOR
DEPARTMENT OF PSYCHOLOGY
SAN DIEGO STATE UNIVERSITY
SAN DIEGO, CA 92120

WERNER, NICOLE E, PHD *
ASSISTANT PROFESSOR
DEPARTMENT OF INDUSTRIAL AND SYSTEMS ENGINEERING
UNIVERSITY OF WISCONSIN-MADISON
MADISON, WI 53706

ZHANG, QI, PHD *
ASSOCIATE PROFESSOR
SCHOOL OF COMMUNITY AND ENVIRONMENTAL HEALTH
OLD DOMINION UNIVERSITY
NORFOLK, VA 23529

## MAIL REVIEWER(S)

MIRZA, SOHAIL K, MD
PROFESSOR AND CHAIR
DEPARTMENT OF ORTHOPAEDICS
DARTMOUTH-HITCHCOCK MEDICAL CENTER
LEBANON, NH 03756

PAZ-SOLDAN, VALERIE ANDREA, PHD
ASSOCIATE PROFESSOR, DIRECTOR OF TULANE HEALTH OFFICES FOR LATIN AMERICA
DEPARTMENT OF GLOBAL COMMUNITY HEALTH AND BEHAVIOR
SCHOOL OF PUBLIC HEALTH AND TROPICAL MEDICINE
TULANE UNIVERSITY
PERU

SHELLEY, DONNA R, MD, MPH
ASSOCIATE PROFESSOR
VICE CHAIR FOR RESEARCH
DEPARTMENT OF POPULATION HEALTH
SCHOOL OF MEDICINE
NEW YORK UNIVERSITY
NEW YORK, NY 10016

WEI, JOHN T, MD
PROFESSOR
DEPARTMENT OF UROLOGY
UNIVERSITY OF MICHIGAN
ANN ARBOR, MI 48109

WYATT, GAIL E, PHD
PROFESSOR
DEPARTMENTS OF PSYCHIATRY AND
BEHAVIORAL SCIENCES
UNIVERSITY OF CALIFORNIA, LOS ANGELES
LOS ANGELES, CA 90024

## SCIENTIFIC REVIEW OFFICER

FERGUSON, YVONNE OWENS, PHD
SCIENTIFIC REVIEW OFFICER
CENTER FOR SCIENTIFIC REVIEW
NATIONAL INSTITUTES OF HEALTH
BETHESDA, MD 20892

## EXTRAMURAL SUPPORT ASSISTANT

BUTLER, SEAN
LEAD - EXTRAMURAL SUPPORT ASSISTANT
CENTER FOR SCIENTIFIC REVIEW
NATIONAL INSTITUTE OF HEALTH
BETHESDA, MD 20892

* Temporary Member. For grant applications, temporary members may participate in the entire meeting or may review only selected applications as needed.

Consultants are required to absent themselves from the room during the review of any application if their presence would constitute or appear to constitute a conflict of interest.
